# Translation machinery reprogramming in programmed cell death in *Saccharomyces cerevisiae*

**DOI:** 10.1038/s41420-020-00392-x

**Published:** 2021-01-18

**Authors:** Francesco Monticolo, Emanuela Palomba, Maria Luisa Chiusano

**Affiliations:** 1 Department of Agricultural Sciences, Università degli Studi di Napoli Federico II, Via Università 100, 80055 Portici, NA Italy; 2 Department of Research Infrastructures for Marine Biological Resources (RIMAR), Stazione Zoologica “Anton Dohrn”, Villa Comunale, 80121 Napoli, Italy

**Keywords:** Transcriptomics, Cell death

## Abstract

Programmed cell death involves complex molecular pathways in both eukaryotes and prokaryotes. In *Escherichia coli*, the toxin–antitoxin system (TA-system) has been described as a programmed cell death pathway in which mRNA and ribosome organizations are modified, favoring the production of specific death-related proteins, but also of a minor portion of survival proteins, determining the destiny of the cell population. In the eukaryote *Saccharomyces cerevisiae*, the ribosome was shown to change its stoichiometry in terms of ribosomal protein content during stress response, affecting the relative proportion between ohnologs, i.e., the couple of paralogs derived by a whole genome duplication event. Here, we confirm the differential expression of ribosomal proteins in yeast also during programmed cell death induced by acetic acid, and we highlight that also in this case pairs of ohnologs are involved. We also show that there are different trends in cytosolic and mitochondrial ribosomal proteins gene expression during the process. Moreover, we show that the exposure to acetic acid induces the differential expression of further genes coding for products related to translation processes and to rRNA post-transcriptional maturation, involving mRNA decapping, affecting translation accuracy, and snoRNA synthesis. Our results suggest that the reprogramming of the overall translation apparatus, including the cytosolic ribosome reorganization, are relevant events in yeast programmed cell death induced by acetic acid.

## Introduction

Programmed cell deaths (PCDs) are a group of diversified processes that are triggered by stress or developmental events, occurring in both prokaryotes and eukaryotes^1–4^. The main phenomenon are associated to the activation of complex molecular pathways that lead to membrane depolarization, DNA fragmentation and breakdown of the plasma membrane^1^. Specific events involving translation processes during PCD have also been reported. In *Homo sapiens*, it has been shown that, during apoptosis, there is a reduction in cap-dependent translation, because of the cap cleavage by caspase 3, or by changes in the phosphorylation state of canonical initiation factors (including eIF4G, eIF4B, eIF4EBP1, and eIF2α)^5,6^. In addition, certain mRNAs, coding for proteins that are essential to the apoptotic process (including Apaf-1^5^ and c-Myc^7^), are translated by the recognition of the internal ribosome entry site (IRES). In *Caenorhabditis elegans* changes in protein synthesis act as a physiological signal to initiate cell death in germ cells. In particular, a modification of the balance between the two eIF4G isoforms (the long and the short ones) disrupts cap-dependent mRNA translation in favor of a cap-independent mechanism. Indeed, the depletion of the long eIF4G isoform promotes the cap-independent translation, inducing the translation of pro-apoptotic proteins^8^. Lastly, in *Drosophila melanogaster*, the pro-apoptotic protein Reaper binds specifically the 40S subunit, affecting the AUG recognition, thereby inhibiting cap-dependent translation^9^, and facilitating the selective translation of specific pro-apoptotic mRNAs thanks to the recognition of IRES segments^10^. Interestingly, the involvement of the translational machinery in *Escherichia coli* PCD has been clearly described by the MazEF module^11^, in the toxin–antitoxin system. Stress conditions (like amino acid starvation, exposure to antibiotics, DNA damage and phage infection, overproduction of ppGpp^12^) reduce MazE levels in the cell, favoring the release of the MazF toxin^13^. This endoribonuclease can produce leaderless mRNAs^14^ and modify 16S rRNA structure in the ribosomes, producing the “stress ribosomes”^15–17^. These events dramatically affect the overall translational process^18^, determining the parallel production of “death” (ClpP, SlyD, YfiD, ElaC, YgcR, and YfbU) and of “survival” (YajQ, RsuA, and DeoC) proteins, promoting a higher propensity to cell death^19^.

*Saccharomyces cerevisiae* (yeast) can undergo PCD triggered by aging, drugs, heat stress, UV radiation, heterologous expression of pro-apoptotic human genes, and acetic acid^20,21^. The process shows the canonical apoptosis hallmarks, such as phosphatidylserine externalization, chromatin condensation and DNA fragmentation, cytochrome *c* release from mitochondria, and dissipation of the mitochondrial transmembrane potential^22^.

The ribosome structure organization in yeast requires a fine-tuning in the gene expression of many of its components, involving ∼200 assembly factors. The 60S ribosomal subunit contains three rRNAs (25S, 5.8S, and 5S) and ∼46 ribosomal proteins, while the 40S subunit is composed of a single rRNA (18S) and ∼33 proteins^23^. The yeast genome is reported to encode 137 ribosomal protein genes (RPGs), ∼150 rDNA repeats, and 76 small nucleolar RNAs (snoRNAs), that are mainly involved in the post-transcriptional modifications of the rRNAs^24,25^. Changes in the expression of each single components participating in the overall process may affect the ribosome biogenesis^25–27^. RPGs in yeast are characterized by couples of paralogs^28^. In particular, among the 137 RPGs annotated in the *S. cerevisiae* genome, 116 were identified to be ohnologs, i.e., duplicated genes, or paralogs, originated by a whole genome duplication^29^. In 2019, Ghulam et al.^30^. demonstrated that the stoichiometry of ohnolog RPGs changes in response to variations of environmental conditions. In particular, under physiological conditions, the ribosome population is dominated by the presence of one of the two paralogs that was defined as the major paralog. When cells are exposed to stress conditions, decreased expression of the major paralog and/or overexpression of the minor one, in ohnolog pairs of ribosomal proteins, reduce the relative proportion of the major versus the minor paralog content in the cell^30^.

We exploited RNA-seq data of yeast undergoing PCD when treated with acetic acid. The experiment considers three different time points after exposure (45, 120, and 200 min)^31^. Our results show changes in the gene expression of RPGs, with differential patterns between pairs of ohnologs when compared to control conditions. Moreover, snoRNA genes are also differentially expressed, revealing additional changes in contributors to ribosomal biogenesis. We show also a differential expression of genes coding for mitochondrial ribosomal proteins (MRPGs). A different trend, moreover, characterizes the expression of the proteins of the mitoribosome versus the ones of the cytosolic ribosome.

This is the first time that changes in ribosome organization are described in yeast PCD, and these events, accompanied by the differential expression of genes involved in the post-transcriptional maturation of the rRNAs and in mRNA translation accuracy, suggest an overall reprogramming of the translation machinery as a hallmark of the early stage of the process.

## Results

### Differentially expressed genes and Gene ontologies

**Fig. 1:**
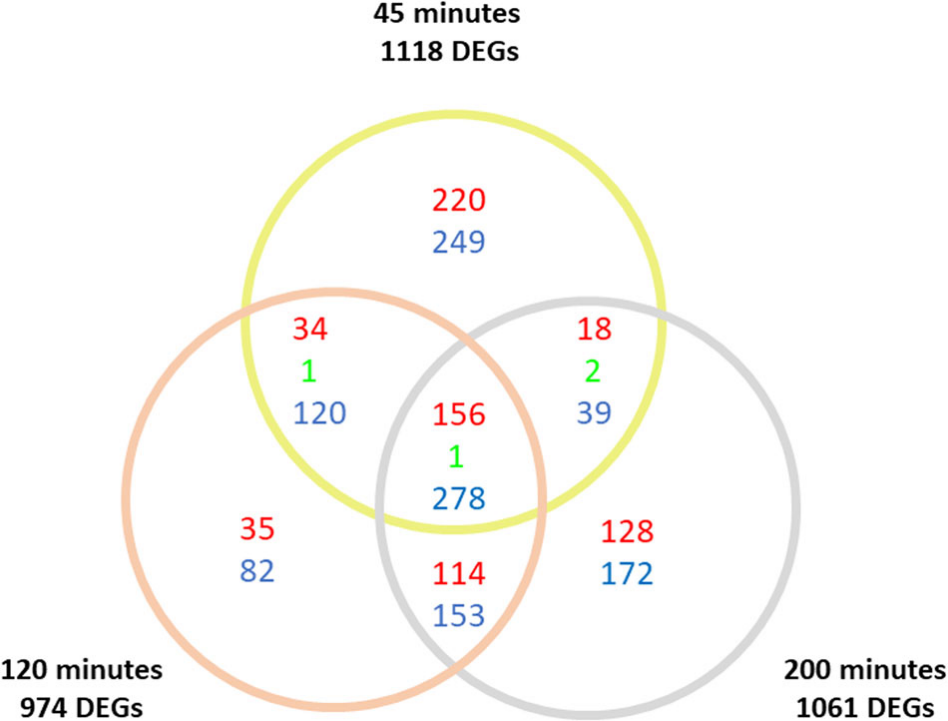
Significant differential expressed genes number per exposure time. Total number of differentially expressed genes (DEGs) and Venn diagram with values at the three time points. In red number of upregulated genes, in blue number of downregulated gene, and in green number of contra-regulated genes.

The processing of the RNA-seq reads concerning the experiments on *S. cerevisiae* exposed to acetic acid^31^ resulted in the list of significant differential expressed genes (DEGs) (Supplemental Table S1) corresponding to 1118 genes at 45 min, 974 at 120 min, and 1061 after 200 min, respectively (Fig. 1). Up and downregulated genes are also indicated, highlighting that among the 435 DEGs in common with the three treatments, 156 resulted always upregulated and 278 were always downregulated in the three experimental time points, whereas one gene (RPL9B) resulted to be upregulated at 45 min, and downregulated at 120 and 200 min (Fig. 1).

**Fig. 2:**
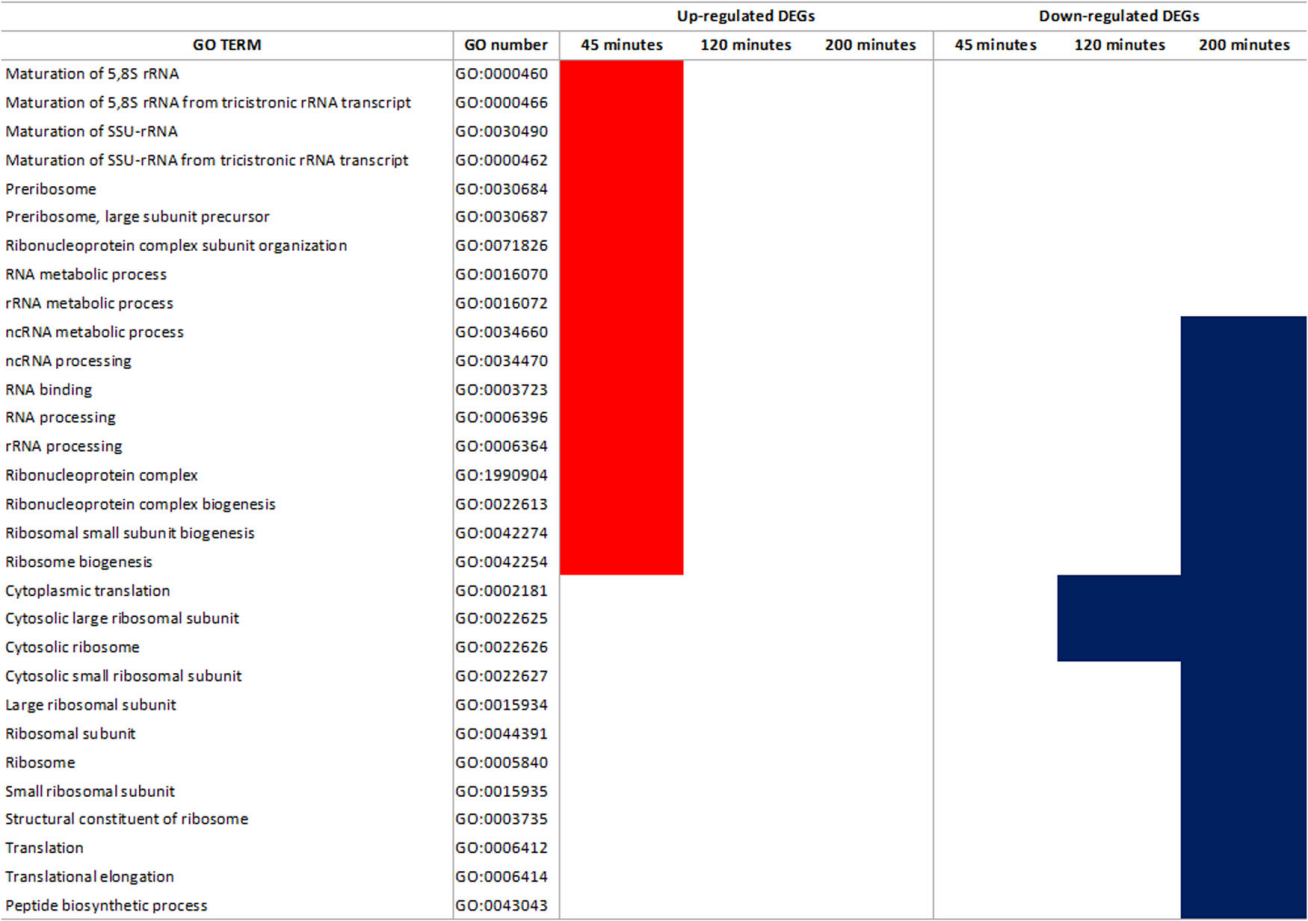
Gene Ontology (GO) enrichment results. List of enriched GOs related to ribosome per exposure time. Colored cells per GO indicate significant enrichment (red for GOs related to upregulated DEGs; blue for GOs related to downregulated DEGs).

The Gene Ontology (GO) enrichment at each time point is in the Supplemental Table S2. In parallel, in Fig. 2 we reported all the enriched GOs related to ribosome organization per time point. The figure shows enrichment by upregulated genes exclusively at 45 min. This occurs for GOs related to rRNA processing and ribosome biogenesis (Fig. 2, Supplemental Table S2). GOs related to cytosolic ribosome and translation are enriched by downregulated genes since 120 min (Fig. 2, Supplemental Table S2). They are accompanied by GO terms related to ribosome biogenesis, rRNA processing, and translation at 200 min (Fig. 2, Supplemental Table S2).

### Functional enrichment

The list of pathways enriched during the three treatments is in the Supplemental Table S3. The pathways enriched with DEGs upregulated at 45 min correspond to cell response to stress and external stimuli. At 120 and 200 min, the enrichment of pathways related to degradation of proteins by the proteasome is evident. The pathways enriched by downregulated DEGs are amino acid metabolism and biosynthesis at 45 min, while ribosomal biosynthesis, ribosomal scanning and start codon recognition and cap-dependent translation initiation pathways are reported at 120 and 200 min (Supplemental Table S3).

### Differential expression of genes involved in mRNA decapping and translation

Considering nuclear encoded genes, it is worthy to note that EDC1 (YGL222C), that is involved in mRNA decapping^32,33^, is upregulated at 200 min (Supplemental Table S1). Moreover, ASC1 (YMR116C), that has been described for its involvement in the control and in the fidelity of the mRNA translational activity^34^, is downregulated at 120 and 200 min (Supplemental Table S1).

### Differential expression of genes involved in ribosome biogenesis

Among the differentially expressed genes, 127 RPGs over 137 total RPGs resulted to be DEGs in at least one stage of the treatments by acetic acid. In particular, at 45 min there are 6 RPGs downregulated and 12 RPGs upregulated. At 120 min and at 200 min there are only downregulated genes, 60 and 122 per stage, respectively (Supplemental Table S1). Interestingly, at 45 min RPL1A (YPL220W) is upregulated whereas its paralog RPL1B (YGL135W) is downregulated. In 2018, Segev et al.^35^ focused on the role of RPL1A and B paralogs. They found that ribosomes containing RPL1B are more efficient in translation of respiration-related proteins, and that this specific form is required for proper mitochondrial function, morphology, and for cell wall integrity. Moreover, they also showed that the upregulation of RPL1A did not replace the functionalities of RPL1B.

A deeper investigation on the differential expression of the RPGs was performed considering all the 137 yeast genes (Supplemental Table S4), where ohnologs^29^ are indicated as consecutive couples. Focusing on gene expression in non-treated samples, an increment in the three-time point of the ribosomal protein gene expression is evident in 134 RPGs (Supplemental Table S4, Supplemental Fig. S1). Only three genes (RPL15A, RPL15B, and RPS14B) slightly decrease their expression at 200 min. The RPGs expression in treated samples shows a completely different pattern. A general decrease of expression is indeed reported in 115 RPGs along the three time points (Supplemental Fig. S1, Supplemental Table S4). Moreover, comparing the expression levels at 45 min, 56 RPGs show a higher expression in treated samples than in non-treated samples (Supplemental Table S4: “yes” in the column log2 CPM Treated/Control >1). It is worth to note that for 30 RPGs the higher expression concerns only one of the two paralogs. At 120 min, in 6 RPGs the expression is higher in treated samples than in non-treated ones and, in all the cases, only one of the two paralogs is involved. At 200 min, in 3 RPGs the expression is still higher in treated samples than in non-treated ones, and, again, all the 3 RPGs involve only one of the two paralogs (Supplemental Table S4). In summary, among the 137 RPGs, 56 have a higher expression in the treatment compared with the relative control experiment, and in 30 out of 38 couples of ohnologs, only one of the two paralogs shows this behavior.

In 2019, Ghulam et al.^30^ classified major and minor paralogs in 20 couples of ohnologs (first 20 couples listed in Supplemental Table S4), among the 58 couples of RPGs ohnologs reported in the yeast genome annotation. Interestingly, they showed that, in general, when cells are exposed to stress, the ratio between the major and the minor paralogs gene expression decreases, because of the downregulation of the major paralog and/or the upregulation of the minor one. In Supplemental Table S4, we highlighted a change in paralogs ratio of 3 of the annotated couples^30^ (“yes” in Paralog couple shift column). Moreover, considering the remaining couples of paralogs, that were not classified in ref. ^30^, the ratio changes also in 8 additional couples. In particular, at 45 min the ratio is changed in 3 couples, at 120 minutes in 4, and at 200 min in 6 couples, respectively (Supplemental Table S4).

### Differential expression of genes involved in mitoribosome biogenesis

Considering the 74 annotated MRPGs, 16 were DEGs in at least one of the treatments using acetic acid (Supplemental Table S1). All these genes are upregulated at 45 min. Whereas, 5 genes (all upregulated) are DEGs after 120 min (Supplemental Table S1). In particular, among the genes upregulated at 45 min, RSM23 (YGL129C) is known to be involved in yeast programmed cell death^36^.

Focusing on all MRPGs expression in non-treated samples, the increment in the three-time points is evident in 61 genes (Supplemental Table S5, Supplemental Fig. S2). Interestingly, in contrast with the general trend reported for cytosolic RPGs (Supplemental Table S4), the MRPGs expression in treated samples shows an increase also in the three stages. Indeed, an increment is reported in 66 genes comparing 120 to 45 min, and in 54 genes comparing 200 to 120 min (Supplemental Table S5). This highlights a peculiar behavior of MRPGs during the exposures to acetic acid.

**Fig. 3:**
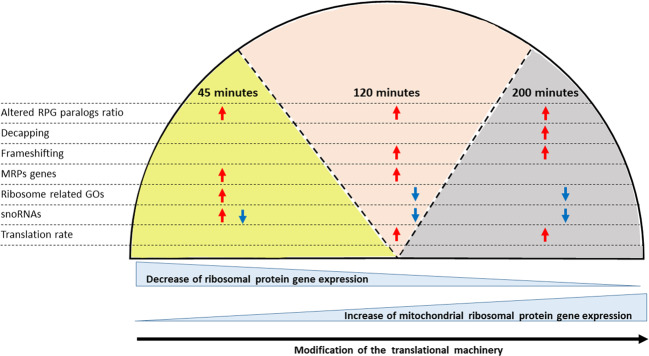
Summary of intracellular changes in protein synthesis machinery induced by acetic acid treatments. Changes after exposure of 45 (light yellow background), of 120 (pink background), and of 200 (gray background) min determine: differential expression of the cytosolic ribosomal protein couples of ohnologs, altering their relative content in the ribosomes (altered ribosomal protein genes (RPGs) ohnologs ratio); differential expression of snoRNAs and of mitochondrial ribosomal protein genes (MRPGs); upregulation of EDC1, encoding an mRNA decapping protein, the downregulation of ASC1, affecting translation rate and frameshifting; enrichment of ribosomal and translation related gene ontologies (GOs). These phenomena are also associated to a general decrease of cytosolic RPGs expression and to the increase of the mitochondrial RPGs expression.

### Differential expression of snoRNAs

Because of their relevance in rRNA post-transcriptional maturation, we analyzed the expression patterns of snoRNAs. Twenty-nine over 77 snoRNAs annotated in the yeast genome are DEGs in at least one treatment. In particular, at 45 minutes, 3 snoRNAs are downregulated and 8 snoRNAs are upregulated; at 120 min, 17 snoRNAs are all downregulated and, at 200 min, 8 snoRNAs are downregulated and 2 snoRNAs are upregulated (Supplemental Table S1).

The snoRNAs are involved in different biological processes including rRNA modifications, maturation, and splicing. Interestingly, although all of them are known to be involved in the ribosome biogenesis, specific roles for some of them have been reported. snR17B, that is revealed upregulated at 45 min, is one of the two box C/D snoRNAs U3 (snR17A, snR17B) that are required for cleavage of the primary rRNA transcript^37^. Moreover, the snR10, snR30 (box H/ACA), and snR128 (box C/D), that are all downregulated at 120 min, are also involved in the cleavage of the primary rRNA transcript^37–39^. Furthermore, loss of snR10 results in slow growth, impaired production of ribosomal small subunits, loss of pseudouridylation at positions 2919 (which occurs in the A loop), and a reduced rate of protein synthesis^26^. This gene is also associated to cells with higher susceptibility to osmotic and cold stresses^25^. In addition, also snR191 is downregulated at 120 min. This H/ACA snoRNA guides pseudouridylation at positions 2258 and 2260 in the large subunit rRNAs. These two modified bases are the most conserved pseudouridines from bacteria to human rRNAs. In yeast, the presence of this snoRNAs and of the conserved pseudouridines is not essential for viability, but it is discussed to provide a small growth advantage in cells placed in competition with cells that lack these features^40^. Finally, also snR5, that is upregulated at 200 min, is reported to be a guide for rRNAs pseudouridylation in eukaryotes^41^. It is assembled with the core domain of Cbf5p, Gar1p, Nop10p, and Nhp2p proteins^42^, and is known to be involved in modifications of uridines, with a relevant role in the process^42,43^.

## Discussion

Acetic acid induces transcriptomic changes^31^ and PCD in yeast^20^. Here we show that transcriptomic changes extensively involve genes associated with ribosome biogenesis and rRNA maturation, also inducing mRNA decapping and affecting translation accuracy, indicating a reprogramming of the cell translational apparatus during the exposure. Indeed, the upregulation of EDC1 (YGL222C), a gene involved in mRNA decapping, highlights the possible role of this mechanism in response to exposure to acetic acid, a phenomena associated also to the yeast response to heat stress^32,33^. In addition, the downregulation of ASC1 (YMR116C), that causes higher translational activity^44^, but favors frameshifting^34^, suggests that the response affects the rate and the quality of the protein synthesis during the response.

We here also show that the exposure to acetic acid induces the differential expression of 127 over 137 RPGs (Supplemental Table S1). Moreover, we highlight that the relative ratio of 11 couples of ribosomal protein ohnologs is changed during acetic acid treatments. This is in agreement with the results of Ghulam et al.^30^, that demonstrated that RPGs ohnologs relative content changes in cells that are exposed to stress. They also show that the repression of the major paralogs and/or the upregulation of the minor paralogs reduce the ratio of the major versus the corresponding minor ohnolog in the cells, increasing the frequency of the minor paralogs in the overall ribosome population. Our results confirm the overexpression of the minor paralogs in 3 of the couples of ohnologs described also in ref. ^30^. This highlights that the changes of the ratios in favor of the minor paralogs occur also in the response to acetic acid (Supplemental Table S4) and suggests that the patterns of changes may be specific of specific responses. Among these, also the two paralogs RPL1A and RPL1B (YPL220W and YGL135W, respectively) have an opposite trend in gene expression at 45 min, with the downregulation of RPL1B and the upregulation of RPL1A, respectively. It is also known that RPL1B is involved in proper mitochondrial function and morphology, roles that cannot be compensated by the paralog RPL1A, when RPL1B is not present in the cell^35^. These effects suggest that the changes in the relative proportion of ribosomal protein ohnologs, that determine the re-organization of the ribosome stoichiometry, and occurring also during acetic acid treatments, may be dramatic for cellular organization and functioning.

Interestingly, also the MRPGs expression is affected by acetic acid treatments (Supplemental Table S1). The gene RSM23 (YGL129C) is upregulated. RSM23 is an ortholog of the human gene DAP-3^45^. DAP-3 was demonstrated to recruit the adaptor protein FADD to the death receptors DR4 and DR5^46^, with its overexpression causing apoptosis in mouse^47^. RSM23 is also involved in yeast programmed cell death. In fact, Madeo et al.^36^ demonstrated that the absence of RSM23 completely prevented induction of apoptosis^36^. This suggests the involvement of the upregulation of a MRP also in acetic acid programmed cell death. Moreover, we here highlight that a typical expression pattern involves other mitoribosomal protein genes when compared with cytosolic ones. The expression of MRPGs does not dramatically change levels when compared with the control, since only 17 of 71 genes resulted significantly upregulated in the first two time points. However, 66 genes over 71 increase their expression levels in the three experimental time points, showing an opposite pattern compared with the one highlighted for cytosolic ribosomal proteins. Indeed, the cytosolic RPGs have a general decrease in gene expression in treated samples when compared with the control. This is also associated with the decrease of expression levels along the three time points after the treatments, in contrast with the constant increase shown in the control experiments.

Our results highlight that transcriptomic changes involve also snoRNAs expression (Table 1). SnoRNAs are involved in the cleavage and in the editing of the primary rRNA transcripts, mainly involving pseudouridylation and methylation^37–39,42,43^. Therefore, the changes in snoRNAs expression levels suggest different patterns of rRNAs structure organization during exposure to acetic acid.

**Table 1:** List of significant differentially expressed snoRNAs.

Differential expression (log2 FC)							
Gene ID	45 min	120 min	200 min	Class	Modification	Target	Position
snR5	1.580	-	1.094	H/ACA box	Pseudouridine	LSU; LSU	1004; 1124
snR8	-	-1.435	-	H/ACA box	Pseudouridine	LSU; LSU	960; 986
snR9	-	-1.780	-1.395	H/ACA box	Pseudouridine	LSU	2340
snR10	-	-2.982	-	H/ACA box	Pseudouridine	LSU	2923
snR11	-	-1.642	-1.277	H/ACA box	Pseudouridine		
snR13	1.049	-	-	C/D box	2′-O-methyladenosine	LSU; LSU	2280; 2281
snR17b	1.180	-	-	Part of small ribosomal subunit processosome			
snR19	-	-	-1.142	U1 spliceosomal RNA			
snR24	-1.106	-	-	C/D box	2′-O-methyladenosine; 2′-O-methylcytidine; 2′-O-methylguanosine	LSU; LSU; LSU	1437; 1449; 1450
snR30	-	-1.769	-	H/ACA box	Pseudouridine	LSU	1109
snR31	-	-1.745	-1.384	H/ACA box	Pseudouridine	SSU	999
snR34	-	-2.254	-	H/ACA box	Pseudouridine	LSU; LSU	2826; 2880
snR36	-	-1.587	-	H/ACA box	Pseudouridine	SSU	1187
snR37	1.351	-	-	H/ACA box	Pseudouridine	LSU	2944
snR39	-	-	-1.528	C/D box	2′-O-methyladenosine	LSU	807
snR42	-	-1.339	-	H/ACA box	Pseudouridine	LSU	2975
snR44	-	-1.438	-1.684	H/ACA box	Pseudouridine	SSUL; SU	106; 1056
snR45	1.345	-2.395	-	C/D box			
snR54	-	-	-1.233	C/D box	2′-O-methyladenosine	SSU	974
snR64	-1.048	-1.936	-	C/D box	2′-O-methylcytidine	LSU	2337
snR67	1.725	-	-	C/D box	2′-O-methylguanosine; 2′-O-methyluridine	LSU; LSU	2619; 2724
snR70	-	-2.586	-1.171	C/D box	2′-O-methylcytidine	SSU	1639
snR81	-	-2.628	-	H/ACA box	Pseudouridine	snRNAU2; snRNAU2	42; 93
snR82	1.612	-	1.559	H/ACA box	Pseudouridine	LSU; LSU; LSU	1110; 2349; 2351
snR86	1.151	-	-	H/ACA box	Pseudouridine	LSU	2314
snR87	-	-1.464	-	C/D box	2′-O-methyladenosine	SSU	436
snR128	-	-1.977	-	C/D box	2′-O-methylcytidine	SSU	414
snR161	-1.102	-	-	H/ACA box	Pseudouridine	SSU; SSU	632; 766
snR191	-	-1.813	-	H/ACA box	Pseudouridine	LSU; LSU	2258; 2260

The log2 fold change (log2FC) of significant differentially expressed snoRNAs are reported per time after exposure (45 min, 120 min, and 200 min). The class (Class), the modification type (Modification), the target (Target), and the position (Position) of each modified rRNA nucleotide are also included.

Changes in the translational machinery are known to occur during stress response or PCD in several species from prokaryotes to eukaryotes. In *E. coli*, the toxin–antitoxin system (mazEF) induces the activation of the toxin MazF under stress response. MazF cleaves single-stranded mRNAs, near the AUG start codon, generating leaderless mRNAs and 16S rRNA at the decoding center. These events lead to the formation of a subpopulation of ribosomes able to translate leaderless mRNAs^18^. In mycobacteria, ribosomes containing the alternative small ribosomal subunit protein RpsR2 generate distinct translational landscapes compared to canonical ribosomes. Moreover, this alternative small ribosomal subunit protein is necessary for growth in iron-depleted medium, highlighting that alternative bacterial ribosomes may play a crucial role during nutrient depletion stress^48^.

In yeast, the formation of a “stress ribosomes” was demonstrated in stress responses, with the involvement of the differential usage of paralogs^30^.

Interestingly, preferential expression of paralogs among ribosomal proteins have also been described in plants. In *Arabidopsis thaliana*, RPS5A is highly expressed in dividing cells, whereas RPS5B is expressed in cells undergoing differentiation^10^. Furthermore, the ribosomal protein RPL11B is highly expressed in all actively dividing cells, whereas the expression of its paralog, RPL11A, is tissue-specific^10^. Moreover, deletion of RPL23AA, but not of RPL23AB, impedes growth and leads to morphological abnormalities^49^.

In *D. melanogaster* two genes encode the ribosomal protein S5, RpS5a and RpS5b. In 2019, Kong et al.^50^ demonstrated that females lacking RpS5b produce ovaries which undergo apoptosis during oogenesis, whereas females lacking RpS5a are fully fertile. This suggested that RpS5b-containing ribosomes are specifically essential during oogenesis^50^.

In *H. sapiens*, ribosomal proteins changes have been demonstrated to be involved in ribosome dysfunctions, leading to ribosomopathies, including Diamond-Blackfan anemia^51^, 5q-syndrome^52^, dyskeratosis congenita, cartilage hair hypoplasia, Treacher Collins syndrome^53,54^, and Shwachman-Diamond syndrome^55^. Moreover, alterations of RPL5, RPL10, RPS15, RPL11, and RPL22 ribosomal proteins have been described in 10 to 40% of tumor types. This highlights that the somatic ribosomal proteins mutations influence the ribosomal functioning, resulting in what is called an oncogenic rewiring of the protein expression mechanisms^56^. In addition, changes in translation mechanisms favoring IRES-dependent protein synthesis where shown in human apoptosis, promoting apoptotic proteins formation^57–60^.

In conclusions, studies from animals, plants and prokaryotes suggest that ribosomal proteins asset plays a crucial role in stress and dysfunctions^61^. Moreover, differential usage of ribosomal protein paralogs resulted to be a remarkable event in stress or dysfunctions, as shown in plants^49^, in *D. melanogaster*^50^, in bacteria^19,48^ and in yeast^30^.

Here we show that the exposure to acetic acid in yeast determines a differential usage of ribosomal protein paralogs, with an overexpression of mitoribosomal proteins. Although more in-depth analyses are required to confirm the compositional modifications at ribosome level, also to shed further light on the molecular mechanisms inducing the differential expression patterns among ohnologs, our analysis highlights that this phenomenon is also accompanied by rRNA modifications, mRNA decapping, affecting translation accuracy and putatively, triggering the synthesis of proteins involved in PCD (Fig. 3). This scenario, here shown for the first time in yeast programmed cell death induced by acetic acid, suggests a possible overall translation reprogramming in eukaryotes cell death.

## Materials and methods

RNA-seq data (fastq files) from *S. cerevisiae* exposed to acetic acid at three different time points (45, 120, and 200 min) plus control data at each stage^31^ were downloaded from sequence read archive (SRA)^62^ (SRP075510). Raw reads were trimmed with Trimmomatic (release 0.38)^63^ and aligned to the *S. cerevisiae* strain S288c genome (version R64-1-1) using STAR (release 2.6.0)^64^ (settings: outFilterScoreMinOverLread=0, outFilterMatchNminOverLread=0, outFilterMatchNmin=0, alignIntronMax=10000, and other parameters as default values). Reads counting was performed using FeatureCounts (release 1.6.3)^65^ (settings: t = “exon”, g = “gene_id”, s = “0”, with the overlapping option and other parameters as default values). Read counts were normalized by counts per million (CPM), filtering genes with CPM ≥ 1 in each replicate per treatment and control. The differential expression analysis was done using edgeR^66^. Only genes with |log_2_Fold change | ≥1 and FDR < 0.05 were considered as significant DEGs.

GO enrichment analysis was performed using YeastMine (https://yeastmine.yeastgenome.org/yeastmine/begin.do)^67^, filtering enriched GOs at *p*-value < 1 × 10^−4^.

Enriched biochemical pathways were obtained by ConsensusPathDB-yeast (http://cpdb.molgen.mpg.de/YCPDB)^68^, filtering enriched pathways at *p*-value < 1 × 10^−4^.

Ohnolog genes in *S. cerevisiae* were downloaded from the Saccharomyces Genome Database (https://www.yeastgenome.org/)^69^ and cross-confirmed by Byrne et al.^29^.

The classification of major and minor paralogs in ohnolog ribosomal protein genes is from Ghulam et al.^30^.

List of snoRNAs and their action (site and type of modification) were downloaded from Modomics (http://genesilico.pl/modomics/)^70^.

## Supplementary information

Supplementary information

Supplemental Figure S1: Ribosomal protein genes expression.

Supplemental Figure S2: Mitochondrial ribosomal protein genes expression.

Supplemental Table S1: List of differential expressed genes.

Supplemental Table S2: Gene ontology enrichment.

Supplemental Table S3: Pathways enrichment.

Supplemental Table S4: Ribosomal protein genes details and paralog couples expression patterns.

Supplemental Table S5: Mitoribosomal protein genes expression patterns.

## References

[1] Galluzzi L (2018). Molecular mechanisms of cell death: recommendations of the Nomenclature Committee on Cell Death 2018. Cell Death Differ..

[2] Carmona-Gutierrez D (2010). Apoptosis in yeast: triggers, pathways, subroutines. Cell Death Differ..

[3] Hengartner MO, Robert Horvitz H (1994). Programmed cell death in *Caenorhabditis elegans*. Curr. Opin. Genet. Dev..

[4] Bayles KW (2014). Bacterial programmed cell death: making sense of a paradox. Nat. Rev. Microbiol..

[5] Spriggs KA, Bushell M, Mitchell SA, Willis AE (2005). Internal ribosome entry segment-mediated translation during apoptosis: the role of IRES-trans-acting factors. Cell Death Differ..

[6] Holcik M, Sonenberg N (2005). Translational control in stress and apoptosis. Nat. Rev. Mol. Cell Biol..

[7] Evan GI (1992). Induction of apoptosis in fibroblasts by c-myc protein. Cell.

[8] Contreras V, Friday AJ, Morrison JK, Hao E, Keiper BD (2011). Cap-independent translation promotes *C. elegans* germ cell apoptosis through Apaf-1/CED-4 in a caspase-dependent mechanism. PLoS One.

[9] Colón-Ramos DA (2006). Direct ribosomal binding by a cellular inhibitor of translation. Nat. Struct. Mol. Biol..

[10] Xue S, Barna M (2012). Specialized ribosomes: a new frontier in gene regulation and organismal biology. Nat. Rev. Mol. Cell Biol..

[11] Aizenman, E., Engelberg-Kulka, H. & Glaser, G. An *Escherichia coli* chromosomal addiction module regulated by guanosine-bispyrophosphate: a model for programmed bacterial cell death. *Proc. Natl Acad. Sci*. **93**, 6059–6063 (1996). 10.1073/pnas.93.12.6059 PMC39188 8650219

[12] Hazan R, Engelberg-Kulka H (2004). Escherichia coli mazEF-mediated cell death as a defense mechanism that inhibits the spread of phage P1. Mol. Genet. Genomics.

[13] Kolodkin-Gal I, Hazan R, Gaathon A, Carmeli S, Engelberg-Kulka H (2007). A linear pentapeptide is a quorum-sensing factor required for mazEF-mediated cell death in *Escherichia coli*. Science.

[14] Zhang Y (2003). MazF cleaves cellular mRNAs specifically at ACA to block protein synthesis in *Escherichia coli*. Mol. Cell.

[15] Metzger S (1988). The nucleotide sequence and characterization of the relA gene of *Escherichia coli*. J. Biol. Chem..

[16] Kolodkin-Gal, I. & Engelberg-Kulka, H. Induction of *Escherichia coli* chromosomal mazEF by stressful conditions causes an irreversible loss of viability. *J. Bacteriol*. **188**, 3420–3423 (2006). 10.1128/JB.188.9.3420-3423.2006 PMC1447462 16621839

[17] Engelberg-Kulka, H., Hazan, R. & Amitai, S. mazEF: a chromosomal toxin-antitoxin module that triggers programmed cell death in bacteria. *J. Cell Sci*. **118**, 4327–4332 (2005). 10.1242/jcs.02619 16179604

[18] Vesper O (2011). Selective translation of leaderless mRNAs by specialized ribosomes generated by MazF in *Escherichia coli*. Cell.

[19] Amitai S, Kolodkin-Gal I, Hananya-Meltabashi M, Sacher A, Engelberg-Kulka H (2009). *Escherichia coli* MazF leads to the simultaneous selective synthesis of both “Death Proteins” and “Survival Proteins”. PLOS Genet.

[20] Ludovico P, Sousa MJ, Silva MT, Leão C, Côrte-Real M (2001). *Saccharomyces cerevisiae* commits to a programmed cell death process in response to acetic acid. Microbiology.

[21] Falcone C, Mazzoni C (2016). External and internal triggers of cell death in yeast. Cell. Mol. Life Sci..

[22] Madeo F (1999). Oxygen stress: a regulator of apoptosis in yeast. J. Cell Biol..

[23] Peña C, Hurt E, Panse VG (2017). Eukaryotic ribosome assembly, transport and quality control. Nat. Struct. Mol. Biol..

[24] Kressler D, Hurt E, Baβler J (2010). Driving ribosome assembly. Biochim. Biophys. Acta.

[25] Ojha S, Malla S, Lyons S (2020). M. snoRNPs: functions in ribosome biogenesis. Biomolecules.

[26] King TH, Liu B, McCully RR, Fournier MJ (2003). Ribosome structure and activity are altered in cells lacking snoRNPs that form pseudouridines in the peptidyl transferase center. Mol. Cell.

[27] Levy S (2007). Strategy of transcription regulation in the budding yeast. PLoS One.

[28] Kellis M, Birren BW, Lander ES (2004). Proof and evolutionary analysis of ancient genome duplication in the yeast *Saccharomyces cerevisiae*. Nature.

[29] Byrne KP, Wolfe KH (2005). The yeast gene order browser: combining curated homology and syntenic context reveals gene fate in polyploid species. Genome Res..

[30] Ghulam, M. M. & Catala, M., & Abou Elela, S. Differential expression of duplicated ribosomal protein genes modifies ribosome composition in response to stress. *Nucleic Acids Res.* **48**, 1954–1968 (2019). 10.1093/nar/gkz1183 PMC7038994 31863578

[31] Dong Y, Hu J, Fan L, Chen Q (2017). RNA-Seq-based transcriptomic and metabolomic analysis reveal stress responses and programmed cell death induced by acetic acid in *Saccharomyces cerevisiae*. Sci. Rep..

[32] Dunckley T, Tucker M, Parker R (2001). Two related proteins, Edc1p and Edc2p, stimulate mRNA decapping in *Saccharomyces cerevisiae*. Genetics.

[33] Neef DW, Thiele DJ (2009). Enhancer of decapping proteins 1 and 2 are important for translation during heat stress in *Saccharomyces cerevisiae*. Mol. Microbiol..

[34] Wolf AS, Grayhack EJ (2015). Asc1, homolog of human RACK1, prevents frameshifting in yeast by ribosomes stalled at CGA codon repeats. RNA.

[35] Segev N, Gerst JE (2017). Specialized ribosomes and specific ribosomal protein paralogs control translation of mitochondrial proteins. J. Cell Biol..

[36] Madeo F (2002). A caspase-related protease regulates apoptosis in yeast. Mol. Cell.

[37] Venema J, Tollervey D (1999). Ribosome synthesis in *Saccharomyces cerevisiae*. Annu. Rev. Genet..

[38] Atzorn V, Fragapane P, Kiss T (2004). U17/snR30 is a ubiquitous snoRNA with two conserved sequence motifs essential for 18S rRNA production. Mol. Cell Biol..

[39] Tollervey D (1987). A yeast small nuclear RNA is required for normal processing of pre-ribosomal RNA. EMBO J..

[40] Badis G, Fromont-Racine M, Jacquier A (2003). A snoRNA that guides the two most conserved pseudouridine modifications within rRNA confers a growth advantage in yeast. RNA.

[41] Ganot P, Bortolin M-L, Kiss T (1997). Site-specific pseudouridine formation in preribosomal rna is guided by small nucleolar RNAs. Cell.

[42] Li S (2011). Reconstitution and structural analysis of the yeast box H/ACA RNA-guided pseudouridine synthase. Genes Dev..

[43] Bortolin ML, Ganot P, Kiss T (1999). Elements essential for accumulation and function of small nucleolar RNAs directing site-specific pseudouridylation of ribosomal RNAs. EMBO J..

[44] Gerbasi VR, Weaver CM, Hill S, Friedman DB, Link AJ (2004). Yeast Asc1p and mammalian RACK1 are functionally orthologous core 40S ribosomal proteins that repress gene expression. Mol. Cell Biol..

[45] Berger, T. et al. The apoptosis mediator mDAP-3 is a novel member of a conserved family of mitochondrial proteins. *J. Cell Sci*. **113**, 3603–3612 (2000). 10.1242/jcs.113.20.3603 11017876

[46] Miyazaki T, Reed JC (2001). A GTP-binding adapter protein couples TRAIL receptors to apoptosis-inducing proteins. Nat. Immunol..

[47] Kissil JL, Cohen O, Raveh T, Kimchi A (1999). Structure–function analysis of an evolutionary conserved protein, DAP3, which mediates TNF-α- and Fas-induced cell death. EMBO J..

[48] Chen Y-X (2020). Selective translation by alternative bacterial ribosomes. Proc. Natl Acad. Sci..

[49] Degenhardt RF, Bonham-Smith PC (2008). *Arabidopsis* ribosomal proteins RPL23aA and RPL23aB are differentially targeted to the nucleolus and are disparately required for normal development. Plant Physiol..

[50] Kong J (2019). A ribosomal protein S5 isoform is essential for oogenesis and interacts with distinct RNAs in *Drosophila melanogaster*. Sci. Rep..

[51] Draptchinskaia N (1999). The gene encoding ribosomal protein S19 is mutated in Diamond-Blackfan anaemia. Nat. Genet..

[52] Ebert BL (2008). Identification of RPS14 as a 5q- syndrome gene by RNA interference screen. Nature.

[53] Liu JM, Ellis SR (2006). Ribosomes and marrow failure: coincidental association or molecular paradigm?. Blood.

[54] Narla A, Ebert BL (2010). Ribosomopathies: human disorders of ribosome dysfunction. Blood.

[55] Boocock GRB (2003). Mutations in SBDS are associated with Shwachman–Diamond syndrome. Nat. Genet..

[56] Sulima SO, Dinman JD (2019). The expanding riboverse. Cells.

[57] Hundsdoerfer, P., Thoma, C. & Hentze, M. W. Eukaryotic translation initiation factor 4GI and p97 promote cellular internal ribosome entry sequence-driven translation. *Proc. Natl Acad. Sci. U.S.A* **102**, 13421–13426 (2005). 10.1073/pnas.0506536102 PMC1224658 16174738

[58] Coldwell MJ, Mitchell SA, Stoneley M, MacFarlane M, Willis AE (2000). Initiation of Apaf-1 translation by internal ribosome entry. Oncogene.

[59] Nevins TA, Harder ZM, Korneluk RG, Holčík M (2003). Distinct regulation of internal ribosome entry site-mediated translation following cellular stress is mediated by apoptotic fragments of eIF4G translation initiation factor family members eIF4GI and p97/DAP5/NAT1. J. Biol. Chem..

[60] Henis-Korenblit S (2002). The caspase-cleaved DAP5 protein supports internal ribosome entry site-mediated translation of death proteins. Proc. Natl Acad. Sci. U.S.A..

[61] Gerst JE (2018). Pimp My Ribosome: ribosomal protein paralogs specify translational control. Trends Genet..

[62] Leinonen R, Sugawara H, Shumway M (2010). & Collaboration, on behalf of the I. N. S. D. The Sequence Read Archive. Nucleic Acids Res..

[63] Bolger AM, Lohse M, Usadel B (2014). Trimmomatic: a flexible trimmer for Illumina sequence data. Bioinformatics.

[64] Dobin A (2012). STAR: ultrafast universal RNA-seq aligner. Bioinformatics.

[65] Liao, Y., Smyth, G. K. & Shi, W. The Subread aligner: fast, accurate and scalable read mapping by seed-and-vote. *Nucleic Acids Res*. **41**, e108 (2013). 10.1093/nar/gkt214 PMC3664803 23558742

[66] Robinson MD, McCarthy DJ, Smyth G (2009). K. edgeR: a bioconductor package for differential expression analysis of digital gene expression data. Bioinformatics.

[67] Balakrishnan, R. et al. YeastMine–an integrated data warehouse for *Saccharomyces cerevisiae* data as a multipurpose tool-kit. *Database* **2012**, bar062 (2012). 10.1093/database/bar062 PMC3308152 22434830

[68] Herwig R, Hardt C, Lienhard M, Kamburov A (2016). Analyzing and interpreting genome data at the network level with ConsensusPathDB. Nat. Protoc..

[69] Cherry JM (2011). *Saccharomyces* genome database: the genomics resource of budding yeast. Nucleic Acids Res..

[70] Boccaletto P (2018). MODOMICS: a database of RNA modification pathways. 2017 update. Nucleic Acids Res..

