# Supplementary material for: Translation machinery reprogramming in programmed cell death in Saccharomyces cerevisiae
Source: Cell Death Discov. 2021 Jan 18;7:17. doi: 10.1038/s41420-020-00392-x (PMC7814045; doi:10.1038/s41420-020-00392-x)
Supplement: Supplementary file 1 — Supplementary information [file 41420_2020_392_MOESM1_ESM.docx]

**Supplementary information**

Supplemental Table S1: List of differential expressed genes.

Gene ID, Gene name, Gene ontology list per gene (GO) and functional annotation (Function) are included. The log2 fold change (log2FC) of significant DEGs at each stage are reported.

Supplemental Table S2: Gene ontology enrichment.

List of GOs enriched at a p-value less than 0.0001 per exposure time. GO number, GO Term, p-value, and the list of genes that are related and therefore enriched the GO term (Gene list) are indicated for down and up regulated significant DEGs.

Supplemental Table S3: Pathways enrichment.

List of pathways (Pathway) enriched by significant down and up-regulated genes at each time point. Reference source database (Source) and p-values (p-value) are also indicated.

Supplemental Table S4: Ribosomal protein genes details and paralog couples expression patterns.

List of ribosomal protein genes (RPGs), their expression as log2CPM in the control and in the treatments at the three exposure times (45 - 120 - 200 minutes), and their classification in terms of paralogy relationships as obtained from Ghulam et al. ^24^ (Paralog classification) are reported (Major and Minor paralogs). Paralogs not described by Ghulam et al. are indicated as “Not defined”, indicating that there is no information confirming the assignment to a Major or a Minor paralog. RPGs with no paralogs are reported as “No paralog”. Significant DEGs and their up or down-regulation are also indicated (up/down). The log2 of the mean of CPMs for the two control samples (log2CPM Control) and the two treated samples (log2CPM Treatment) are also shown. Genes with log2CPM Treated/Control Ratio >1 are marked by “yes”. The two ratios between log2CPMs of couple of paralogs are shown in control and treatment samples (log2 CPM paralog ratio Control and log2 CPM paralog ratio Treatment, respectively) to show the ratio and indicate the shift among paralogs (Paralog couple shift = yes/not).

Supplemental Table S5: Mitoribosomal protein genes expression patterns.

List of mitoribosomal protein genes and their expression at the three time points (45 - 120 - 200 minutes) indicated as log2 of the mean of CPMs for the two control samples (log2CPM Control) and the two treated samples (log2CPM Treatment). Significant DEGs and their up or down-regulation are also indicated.

Supplemental Figure S1: Ribosomal protein genes expression.

Bar plots highlighting the RPGs expression (reported as CPMs) and the patterns in control samples at each stage (blue), and in treated samples at each stage (45 minutes (red), 120 minutes (yellow) and 200 minutes (green)).

Supplemental Figure S2: Mitochondrial ribosomal protein genes expression.

Bar plot highlighting the mitoribosomal protein genes expression (as CPMs) and the patterns in control samples, at each stage (first three blue lines per gene), versus treated samples, lines 4, 5 and 6 per gene ((45 minutes (red), 120 minutes (yellow) and 200 minutes (green)).
