# Supplementary figures and images for: Translation machinery reprogramming in programmed cell death in Saccharomyces cerevisiae
Source: Cell Death Discov. 2021 Jan 18;7:17. doi: 10.1038/s41420-020-00392-x (PMC7814045; doi:10.1038/s41420-020-00392-x)

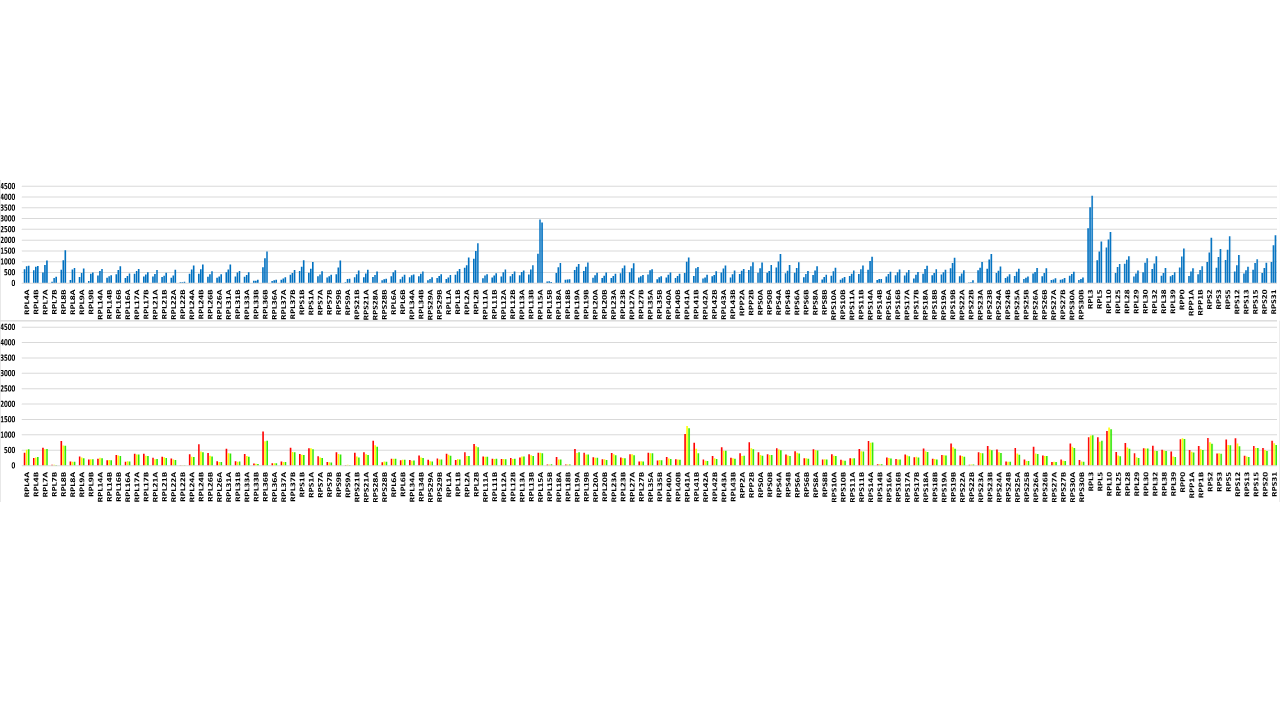

Supplement: Supplementary file 2 — Supplemental Figure S1: Ribosomal protein genes expression. [file 41420_2020_392_MOESM2_ESM.tif]

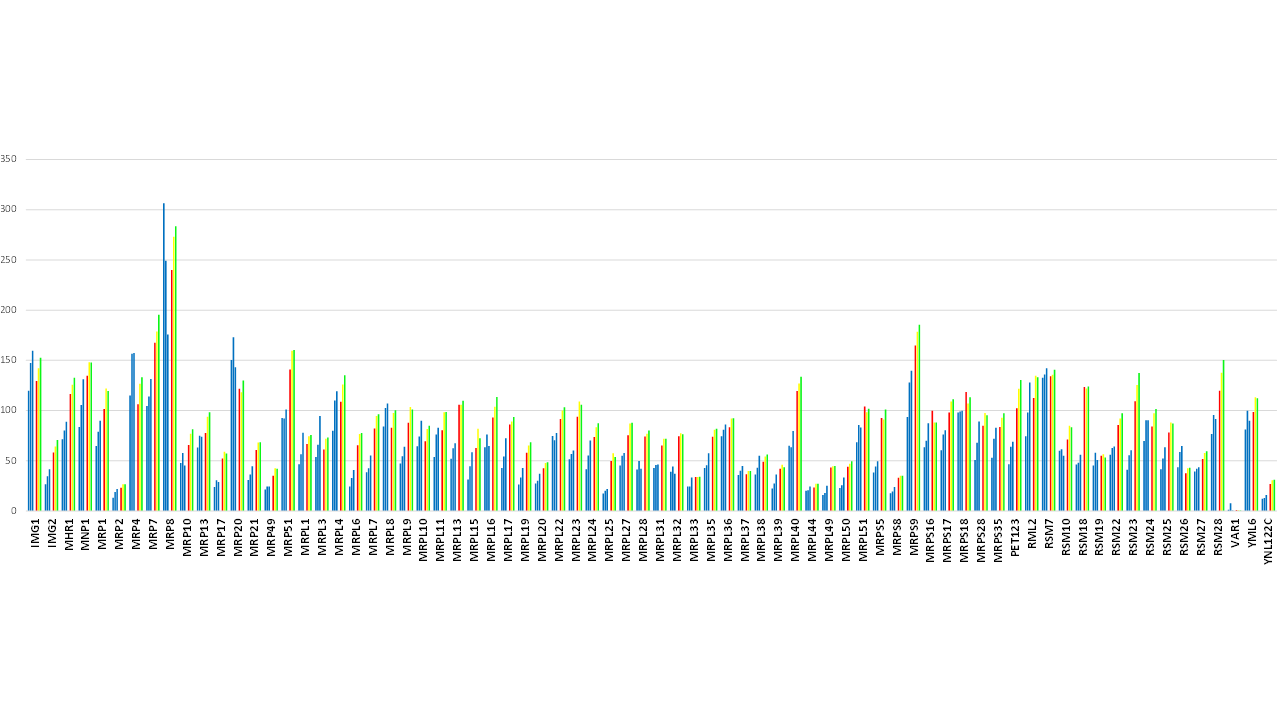

Supplement: Supplementary file 3 — Supplemental Figure S2: Mitochondrial ribosomal protein genes expression. [file 41420_2020_392_MOESM3_ESM.tif]
